# First Detection of *Ditylenchus destructor* Parasitizing Maize in Northeast China

**DOI:** 10.3390/life11121303

**Published:** 2021-11-26

**Authors:** Fengjuan Pan, Feng Li, Yanzhi Mao, Dan Liu, Aoshuang Chen, Dan Zhao, Yanfeng Hu

**Affiliations:** 1 Northeast Institute of Geography and Agroecology, Chinese Academy of Sciences, Harbin 150081, China; panfj@iga.ac.cn; 2 Syngenta (China) Investment Co., Ltd., Beijing 102206, China; feng.li@syngenta.com (F.L.); Daniel.liu@syngenta.com (D.L.); 3 Heilongjiang Academy of Agricultural Sciences, Harbin 154026, China; kshpotato@163.com (Y.M.); zd1978722@163.com (D.Z.); 4 College of Advanced Agriculture and Ecological Environment, Heilongjiang University, Harbin 150006, China; nixuerong21@mails.ucas.ac.cn

**Keywords:** *Ditylenchus destructor*, plant-parasitic nematodes, morphological characteristic, maize

## Abstract

Maize is one of the most important crops in the world. Heilongjiang province has the largest maize area in China. Plant-parasitic nematodes are important agricultural pests, which cause huge economic losses every year and have attracted global attention. Potato rot nematode *Ditylenchus destructor* is a plant-parasitic nematode with a wide range of hosts and strong survival ability in different environments, which brings risks to agricultural production. In 2020, *D. destructor* was detected in seven maize fields in Heilongjiang province. Morphological identification and molecular approach were used to characterize the isolated *D. destructor*. The observed morphological and morphometric characteristics were highly similar and consistent with the existing description. The DNA sequencing on the D2/D3 region of the ribosomal DNA 28S and the phylogenetic analysis showed that *D. destructor* population obtained from maize and other isolates infesting carrot, sweet potato, and potato were in subclade I supported by a 96% bootstrap value. Additionally, the phylogenetic analysis of the ITS rRNA gene sequence further indicated that this *D. destructor* population from maize clustered in a clade I group and belonged to ITS rRNA haplotype C. An inoculation experiment revealed that *D. destructor* was pathogenic on the maize seedlings in pots and caused the disease symptoms in the stem base of maize seedlings. This is the first report of *D. destructor* causing stem rot of maize in Heilongjiang province, and contributes additional information on disease control and safe production of maize in the region.

## 1. Introduction

Maize (*Zea mays* L.) is one of the most important foods and sustains millions of people worldwide [1]. According to Food and Agriculture Organization of the United Nations (FAO) estimates, in 2019, over 1.97 × 10^8^ ha of maize were planted, and more than 1.15 × 10^9^ tons of maize were produced worldwide [2]. China is the largest producer of maize in the world, with an estimated 4.13 × 10^7^ ha of planted area [2].

Plant-parasitic nematodes (PPNs) are some of the most difficult pests to control, causing an estimated USD 173 billion in global yield loss each year [3]. The disease caused by PPNs has attracted global attention. Many PPN nematode species have been reported to seriously affect the production of field crops. For example, *Heterodera glycine* contributes to soybean cyst nematode disease [4], *Heterodera avenae* causes wheat cyst nematode disease [5], *Aphelenchoides besseyi* causes rice white-tip nematode disease [6], and *Heterodera zeae* causes maize cyst nematode disease [7].

Some PPNs, such as *Meloidogyne*, are considered serious pests of economically important vegetables and crops [8]. The potato rot nematode, *Ditylenchus destructor*, is an economically important pathogenic nematode in agriculture. In previous studies, *D. destructor* primarily infected and damaged sweet potato (*Ipomoea batutas*), potato (*Solanum tuberosum*) [9,10], and other plants with tubers and bulbs [11]. *D. destructor* can cause an average of 10% yield loss of potato crops in the USA [12]. In China, *D. destructor* has been one of the major restrictive factors for production of sweet potato. In recent years, this plant-parasitic nematode has become the most serious pathogen of sweet potato in northern China, inducing 20% to 50% yield loss, sometimes even 100% [13]. The nematode also infects angelica, ginseng, notoginseng, and other herbs and can induce yield loss in angelica as high as 84.9% [11]. *D. destructor* is a migratory endoparasitic nematode, and many countries have listed this species as a plant quarantine nematode [14].

In China, the research on maize diseases mainly focused on fungal diseases, such as *Setosphaeria turcica* [15], *Bipolaris maydis* [16], and *Sporisorium reilianum* [17]. For PPN-induced maize damage, *Trichotylenchus changlingensis* caused the maize nematode stunt disease in Northeast China [18]. *Heterodera* *elachista* was reported to infect maize in Italy [19] and northwest China [20]. *Heterodera* *zeae* parasitizing maize was found in Guangxi Province of China [21].

**Figure 1:**
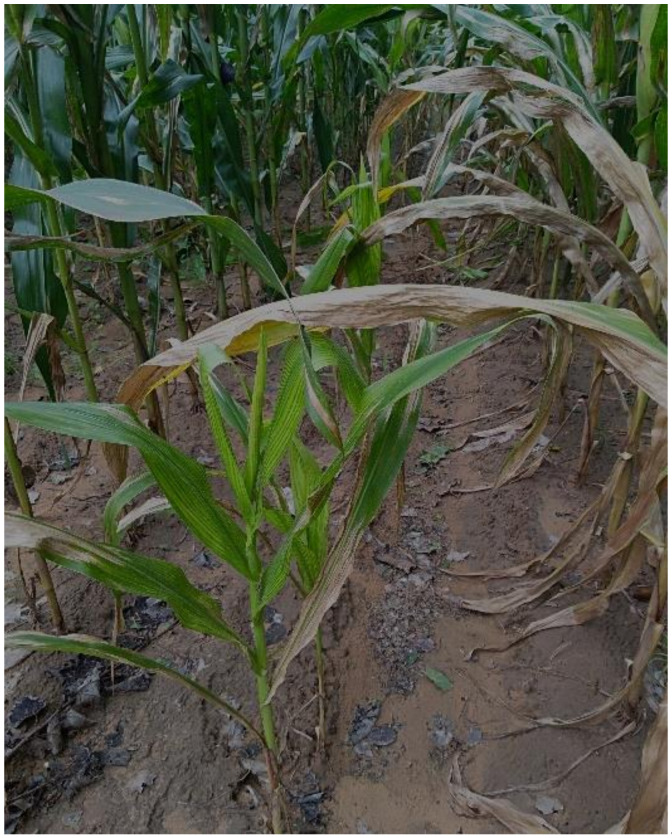
Maize plants with *Ditylenchus destructor* damage in the field.

In 2020, *D. destructor* was identified from maize roots and rhizosphere soil in maize fields in northeast China, where the maize plants were shorter and thinner than expected (Figure 1). Although previous studies have reported that maize may be one of the hosts of *D. destructor* [22] and potato was infected by *D. destructor* in Heilongjiang province [23], this is the first observation of damage caused by *D. destructor* in maize fields in Heilongjiang province. This is a potential pathogenetic nematode that can cause serious damage to maize production. The aim of the present study was to analyze the morphological, morphometric, and molecular characteristics of *D. destructor* isolates from the maize.

## 2. Materials and Methods

### 2.1. Soil Sampling

During the 2020 survey to determine plant-parasitic nematode populations in Heilongjiang province, top soils of 0–15 cm were collected in maize fields with a shovel. Each soil sample was a composite of five sub-samples taken in a 20 m × 20 m area. Soil samples were sieved (1 cm mesh) to move the plant roots and large stones. A total of 202 soil samples (500 g per soil sample) were collected, placed in polyethylene bags and brought to the laboratory for soil nematode extraction.

### 2.2. Nematode Extraction and Observation

Soil nematodes were extracted from fresh soils for 48 h using the modified Baermann tray method [24]. The nematodes were heat-killed at 60 °C for 10 min and preserved in a 4% formaldehyde solution. The nematodes in each sample were observed and counted using an anatomical lens (SZX16, Olympus, Tokyo, Japan) and specimens suspected to be *D. destructor* were further observed under microscope (BX-43F, Olympus, Tokyo, Japan).

### 2.3. Morphological and Morphometric Characterization of D. destructor

Permanent specimens of *D. destructor* were made for long-term preservation and observation using a glycerol–alcohol dehydrating process [25], and then observed and measured under microscope. Females and males of *D. destructor* were selected under the anatomical lens and transferred to a small petri dish (diameter 3 cm) with 1 mL dehydrated liquid I. The small petri dish was placed in a desiccator with 95% alcohol for about 12 h at 40 °C. *D. destructor* was selected under the anatomical lens and transferred to the small petri dish with dehydrating liquid Ⅱ. The small petri dish was placed in a drying oven for about 3 h at 40 °C, allowing the dehydrating liquid Ⅱ to evaporate slowly. The dehydrated *D. destructor* was transferred in a drop of glycerin on a glass slide, covered by a cover glass, and sealed with paraffin by heating it over a spirit lamp. The sealed *D. destructor* was photographed and measured using a microscope (DM2500, Leica, Wetzlar, Germany).

### 2.4. Molecular Identification of D. destructor

Genomic DNA was extracted from 10 hand-picked nematodes including females, males and juveniles according to previous studies [26]. Nematodes were washed with sterile ddH_2_O three times in a 0.2 mL PCR tube, then an equal volume (10 μL) lysis buffer containing 10 mM Tris-HCL (pH 8.0), 50 mM KCl, 2.5 mM MgCl_2_, 0.5 mM EDTA, 1 mM dithiothreitol, 60 mg/mL proteinase-K, and 0.45% Tween 20 was added. Nematode suspensions were immediately immersed in liquid nitrogen for 30 min. Lysis reaction took place in a Bio-Rad thermocycler (Hercules, CA, USA) with an initial step at 65 °C for 90 min and a final step at 95 °C for 10 min. Lysate was briefly centrifuged and stored at −20 °C for later use.

The D2/D3 region of 28S rRNA and the internal transcribed spacer (ITS)-rRNA were amplified using two sets of universal primers D2A/D3B (5′-ACAAGTACCGTGAGGGAAAGTTG-3′; 5′-TCGGAAGGAACCAGCTACTA-3′) [27] and rDNA1/rDNA2 (5′-TTGATTACGTCCCTGCCCTTT-3′; 5′-TTTCACTCGCCGTTACTAAGG-3′) [28], respectively. The PCR reaction was performed in a total volume of 50 μL containing 10 μL 5× SF Buffer (with 10 mM MgSO_4_), 1 μL Phanta Super-Fidelity DNA Polymerase (Vazyme, Nanjing, China), 1 μL dNTP Mix (10 mM each), 2 μL forward and reverse primers (10 μM), and 2 μL template DNA. The cycling conditions were as follows: 95 °C for 3 min, followed by 35 cycles consisting of denaturation at 95 °C for 10 s, annealing at 55 °C for 20 s, and extension at 72 °C for 30 s, with a final extension at 72 °C for 10 min. The PCR products were electrophoresed through a 1% agarose gel and further purified using Thermo Scientific GeneJET PCR purification kit (Thermo Fisher Scientific, Waltham, MA, USA). Amplified fragments were cloned into pLB fast cloning vector (TIANGEN, Beijing, China) and sequenced by TSINGKE Biological Technology (Harbin, China). Newly obtained sequences of *D. destructor* in this study were sent to GenBank (GenBank accession no. MT585824 and OL373915). The sequences of PCR product as a query were blasted in the NCBI gene database for similarity search, and multiple relevant sequences were obtained from the database for alignment.

Multiple alignments of sequence obtained in the present study and those retrieved from GenBank were conducted using ClustalX 1.81 [29]. A phylogenetic tree resulting from analysis of the D2/D3 region of 28S rRNA was constructed using MEGA X software [30] with the maximum likelihood (ML) method based on the Kimura 2 parameter model. A bootstrap test was performed to determine statistical consistency of each branch using a bootstrapped dataset with 1000 cases. The phylogenetic tree of the full ITS (ITS1-5.8S-ITS2) was obtained by Bayesian inference using Mrbayes3.04. The HKY + F model was selected as the best-fit model of DNA evolution using ModelFinder. Bayesian analysis was initiated with a random starting tree and run with the Metropolis-coupled Markov chain Monte Carlo for 2 × 10^6^ generations. Two sequences from *Ditylenchus myceliophagus* (DQ151458.1 and AM232236.1) were used to construct the outgroup of the phylogenetic tree. Posterior probability more than 75% was given for appropriate clades.

### 2.5. Pathogenicity Verification of D. destructor

Soil nematodes were extracted from the soil samples containing *D. destructor*. The active individuals of *D. destructor* were selected under the anatomical lens, and washed three times with sterile water. The nematodes were re-suspended in a sterile antibiotic solution containing 0.01% mercuric chloride and 0.002% sodium azide for 10 min, and then immediately washed four times in sterile water. The sterile *D. destructor* was inoculated on the surface-sterilized carrot callus in sterilized upper clean bench, and incubated in the dark for 30 days at 20–25 °C for multiplication.

Maize seeds (Limin 33) were germinated on moist paper, and germinated seeds of uniform size were hand-picked. The seed surfaces were sterilized with 2% NaOCl for 10 min and then rinsed three times with sterile water. Maize seeds were germinated at room temperature (about 23 °C). Maize seeds with a bud length of 0.5 cm to 1 cm were sown into 250 mL paper cups (one seed per cup) containing sterilized mixture of soil and sand (4:1). The nematodes were collected from carrot disc callus with 25 μm mesh sieve. A suspension of *D. destructor* (approximately 1500 individuals) was inoculated into each paper cup, while the same amount of water was applied to the paper cup for the control treatment. After 14 days since inoculation, maize plants and soils were transferred to pots (12 cm diameter × 10 cm deep) with sterilized soil. The maize was pulled out 30 days after inoculation. The soils on the roots were washed to observe the damage symptoms on the maize stem base. *Ditylenchus destructor* in the stem base was stained with acid fuchsin and observed under a stereoscope.

## 3. Results and Discussion

### 3.1. Morphological and Morphometric Characteristics

Morphological characteristics and morphometric studies were performed from the recovered females and males of the isolate of *D. destructor* (Table 1).

**Figure 2:**
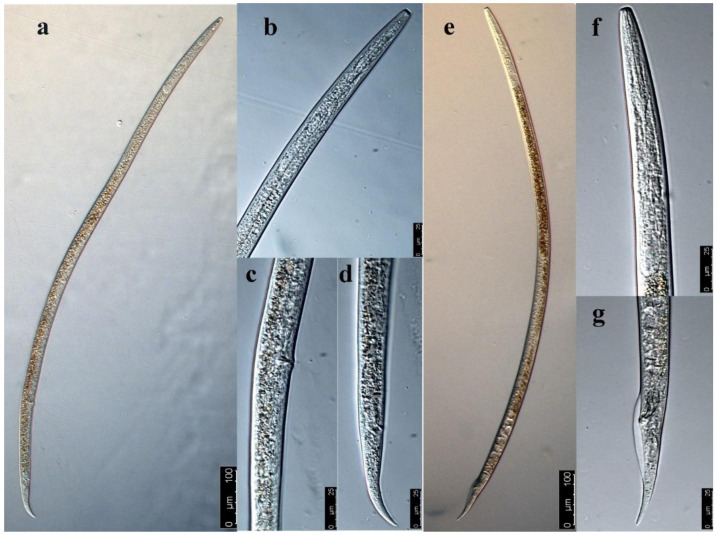
The morphological characteristics of *Ditylenchus destructor* under microscope. (**a**) Female, (**b**) head of female, (**c**) vulva of *D. destructor*, (**d**) tail of female, (**e**) male, (**f**) head of male, (**g**) tail of male.

The female body was elongated. The nematode body was straight, with tail slightly bent towards its abdomen after being killed by 60 °C hot water (Figure 2a). The lip region was continuous with body contour, with an anteriorly flat surface (Figure 2b). The stylet was well developed with two almost equal parts, with a rounded cone and distinct basal knobs. The valvulate metacorpus was a long spindle shape, accounting for about 1/3 of body width. The posterior esophagus part was overlapped dorsally with the intestine. The excretory pore was located at the junction of the intestine and esophagus and slightly toward the anterior of the body. The female genital system was anteriorly outstretched. The post-uterine sac was long and often extended to about 3/4 of the vulva–anus distance (Figure 2c). The vagina was deep and slightly protuberant. The tail was conical with rounded terminus (Figure 2d).

The male body was slenderer than the female body, and the anterior morphology was similar to that of the female (Figure 2e,f). The single testis extended forward, and the front end reached esophagus gland base. The spicule was ventrally curved, and its base was enlarged. The gubernaculum was short and did not extend beyond the cloaca. The wide bursa extended to near the middle of the tail (Figure 2g).

Morphological identification is a valuable approach for *D. destructor*, which is restricted to preferably adult specimens. The morphology and morphometrics of *D. destructor* were compared to the description of those isolated from nematodes infecting potato [31], *Codonopsis pilosula* [32], and dahlia [33]. Our results were consistent with previous descriptions (Table 1). However, the present study indicates that host plants may cause minor variations in characteristics of *D. destructor,* such as the body size and spear size. Identification of *D. destructor* species based on morphological characteristics remains a challenge, as morphological differences between *Ditylenchus* species are not apparent in most cases and the morphological characteristics of different nematode species generally overlap. For example, the morphological characteristics of *D. destructor* are similar to those of *D. convallariae*, and the morphometrics (nematode body length, style length, ratio of body length to maximum body width, ratio of distance between head and vulva to body length) overlap [34]. Similarity between *Ditylenchus* species brings great difficulties for the beginner and non-professional workers of nematode identification. The morphological characteristics of nematodes also vary regionally, especially the size of nematodes, which is affected by climate and host species [35,36]. Therefore, morphological identification of *D. destructor* needs to be supported by molecular techniques.

### 3.2. Molecular Characterization

**Figure 3:**
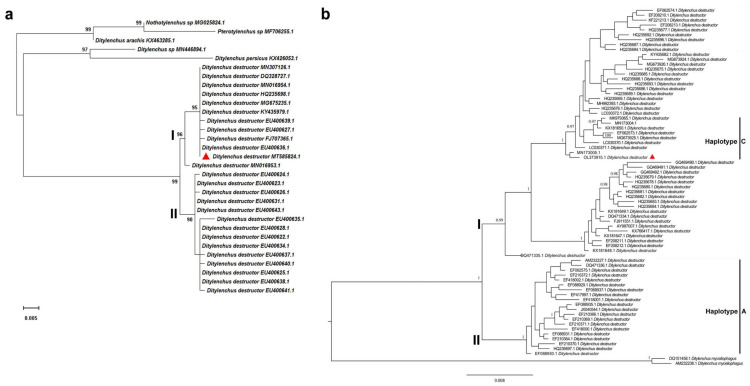
(**a**) Maximum likelihood (ML) analysis of the D2/D3 region of 28S rRNA sequence of Ditylenchus destructor isolates in this study with other reference sequences based on the Kimura 2-parameter model. Analysis was performed using 1000 bootstrap replicates. (**b**) Phylogenetic tree of ITS-rRNA gene sequences from Ditylenchus destructor populations constructed using Bayesian analysis. The sequences were analyzed using the HKY + F substitution model. Posterior probability more than 75% is given for appropriate clades. Newly obtained sequence from Ditylenchus destructor popu-lation is indicated by the red triangle.

The PCR amplification of the D2/D3 fragments of the 28S rRNA and the ITS-rRNA region for nematodes infecting maize plants yielded two DNA fragments of approximately 780 bp and 1130 bp, respectively. The nucleotide sequences obtained in this study were deposited into the GenBank database (accession no. MT585824 and OL373915). A BLAST search of the nucleotide sequence of the D2/D3 was conducted and showed a similarity of 98.8–100% with multiple sequences of *D. destructor* populations available in the GenBank database. Among them, the top three sequences with highest identity (100%) are EU400636 (Query Cover = 100%, E value = 0.0), MG675235 (Query Cover = 99%, E value = 0.0), and KY435979 (Query Cover = 99%, E value = 0.0), which are three *D. destructor* isolates from China. The phylogenetic analysis based on the maximum likelihood method further revealed that the *D. destructor* population obtained from maize and other isolates of *D. destructor* infesting carrot, sweet potato, and potato from China and other countries are in one clade supported by a 99% bootstrap value (Figure 3a). The ITS-rRNA region of *D. destructor* from maize contained an 845 bp full length of ITS1-5.8S-ITS2 region and had 99.4–100% identity with five known sequences of *D. destructor* (MK979365.1, MZ345883.1, MN173004, MN173005, EF208210, and KF221213) infecting other plants. Bayesian analysis of the ITS1-5.8S-ITS2 rRNA gene sequences of *D. destructor* populations generated a phylogenetic tree with two main clades (Figure 3b). Phylogenetic relationship showed that this *D. destructor* population from maize and other reported populations [37] was clustered in Clade Ι and belonged to the ITS haplotype C.

In the process of biological evolution, the D region of 28S rDNA has been considered as an abundant and conserved structure for sequence diversity study [38,39]. In particular, the D2/D3 region is widely used in the classification of nematode genus and species and the identification of related species. For example, the D2/D3 region of 28S rDNA was used to study the phylogenetic relationships of *Meloidogyne*, *Pratylenchus*, *Radopholus*, *Hirschmanniella*, *Helicotylenchus*, and *Rotylenchus* [40,41,42,43]. The D2/D3 region was also used for species identification of *Bursaphelenchus* and *Meloidogyne* [44,45] and phylogenetic analysis of *D. destructor* [32,46]. The D2/D3 region of 28S rDNA contains phylogenetic information which can provide important basis for phylogenetic comparison and identification of PPNs. In this study, we successfully identified nematodes isolated from maize field using the sequence of the D2/D3 region, and our ML analysis of the D2/D3 sequences generated a tree with the largest clade including all *D. destructor* populations from different host plants. This clade was subdivided into two subclades, and subclade Ⅰ was composed of our *D. destructor* samples and other *D. destructor* populations mainly from sweet potato and potato. A similar topology tree with two main clades was also reported in a previous study through Bayesian analysis of the D2/D3 of 28S rDNA of *D. destructor* populations [37]. Several studies showed that the extensive length variation and minisatellite internal repeats are present in the ITS-rRNA region of *D. destructor* [37,47], which suggested that the ITS region is a good marker for population genetics and biogeographical studies of *D. destructor*. Previously, the haplotypes of the ITS1-5.8S-ITS2 rRNA gene of *D. destructor* populations were designated two ITS haplotypes (A and B) [48]. Analysis of the ITS sequence divergences by Subbotin et al. further revealed that *D. destructor* populations are clustered in two main clades (Ⅰ and Ⅱ) containing seven haplotypes (A, B, C, D, E, F, and G), and all of them are found in China except for type G [37]. A recent study reported that the *D. destructor* populations parasitic in potatoes belonged to the ITS haplotype C [31]. In the present study, we found that the *D. destructor* ITS-rRNA region from maize showed the 99.6–100% identity with those ITS-rRNA sequences (MK979365.1, MN173004, and MN173005) of *D. destructor* populations from potatoes [31]. Additionally, phylogenetic tree analysis further indicated that the *D. destructor* in maize field belonged to clade Ⅰ and haplotype C. Molecular techniques have been widely used for identification of plant-parasitic nematodes. However, due to the genetic closeness between nematode species, molecular identification needs to be used in combination with morphological and morphometric characterization.

### 3.3. Pathogenicity Verification of D. destructor

**Figure 4:**
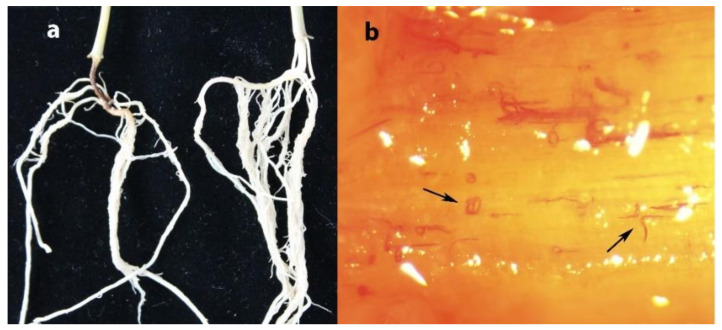
(**a**) Maize root infected by *D. destructor* indicated by the arrow, (**b**) stained *Ditylenchus destructor* in stem base of maize.

A total of 202 soil samples were collected in maize fields in Heilongjiang province in 2020, and *D. destructor* was detected in seven soil samples, with an occurrence frequency of 3.47% (data are not shown). After 30 days of *D. destructor* infection, brown disease symptoms appeared on the stem base of the maize seedling near the ground (Figure 4a, left seedling). When maize seedings were severely damaged, the stem base turned brown, shrunk, and eventually collapsed. The adults and juveniles of *D. destructor* were observed in the stained stem base under anatomical microscope (Figure 4b).

A previous study found that potato rot disease occurred only slightly on the potatoes that were planted consecutively in some potato fields with severe potato rot in the previous year, while serious potato rot disease occurred in the first year when potato was planted after long-term continuous cropping of wheat and maize [22]. This may be due to the fact that *D. destructor* was taken away with disease tissues after the potato was harvested, and the population of the nematode in the soil was small. Some maize seedlings were seriously infected by *D. destructor* and eventually died, but farmers did not recognize nematodes as causing the problem and may have attributed the damage to other diseases or factors. Low-density *D. destructor* populations may cause undetectable damage to maize, causing neglect of this pathogen. This neglect will pose a potential threat to future agricultural production as populations may increase, and *D. destructor* may evolve to better adapt to maize as a new host.

In general, once *D. destructor* has entered the host plants, it is difficult to control even with the application of excessive pesticides. At present, pretreatment of soil with low-toxicity nematicide and biological control are more suitable methods for the prevention and control of *D. destructor* [49]. In China, *D. destructor* occurs in 12 provinces [22]. It is necessary to survey the density of *D. destructor* populations before sowing, especially for potato and sweet potato. In this study, a pot inoculation experiment confirmed the pathogenicity of *D. destructor* to maize. To our knowledge, this is the first report of *D. destructor* in maize field in Heilongjiang province, which provides valuable information for the production of maize.

## Figures and Tables

**Table 1:** Morphological characteristics of the females and males of the isolate of *D. destructor*. Measurements are presented as a range.

	Maize (n = 40)	
	Female	Male
L (μm)	801.7–1009.5	795.1–907.5
a	25.4–36.5	31.2–41.5
Stylet (μm)	8.9–12.6	10.1–12.2
b	6.2–7.0	5.5–7.0
c	10.4–16.0	10.9–13.8
c’	3.3–4.8	3.8–5.7
v	76.2–80.0	
Spicule length (μm)		21.6–26.7

## Data Availability

Sequencing data reported in this study were deposited to GenBank available online at https://www.ncbi.nlm.nih.gov/genbank/ (accessed on 22 November 2021).
